# Understanding and predicting pregnancy termination in Bangladesh: A comprehensive analysis using a hybrid machine learning approach

**DOI:** 10.1097/MD.0000000000038709

**Published:** 2024-06-28

**Authors:** Riaz Rahman, Ashis Talukder, Shatabdi Das, Joy Saha, Haribondhu Sarma

**Affiliations:** a Statistics Discipline, Science Engineering and Technology School, Khulna University, Khulna, Bangladesh; b Applied Epidemiology, National Centre for Epidemiology and Population Health, Australian National University, Acton, ACT, Australia.

**Keywords:** Bangladesh, hybrid model, pregnancy termination, risk factors

## Abstract

Reproductive health issues, including unsafe pregnancy termination, remain a significant concern for women in developing nations. This study focused on investigating and predicting pregnancy termination in Bangladesh by employing a hybrid machine learning approach. The analysis used data from the Bangladesh Demographic and Health Surveys conducted in 2011, 2014, and 2017 to 2018. Ten independent variables, encompassing factors such as age, residence, division, wealth index, working status, BMI, total number of children ever born, recent births, and number of living children, were examined for their potential associations with pregnancy termination. The dataset undergoes preprocessing, addressing missing values and balancing class distributions. To predict pregnancy termination, 8 classical machine learning models and hybrid models were used in this study. The models’ performance was evaluated based on the area under the curve, precision, recall, and F1 score. The results highlighted the effectiveness of the hybrid models, particularly the Voting hybrid model (area under the curve: 91.97; precision: 84.14; recall: 83.87; F1 score: 83.84), in accurately predicting pregnancy termination. Notable predictors include age, division, and wealth index. These findings hold significance for policy interventions aiming to reduce pregnancy termination rates, emphasizing the necessity for tailored approaches that consider regional disparities and socioeconomic factors. Overall, the study demonstrates the efficacy of hybrid machine learning models in comprehending and forecasting pregnancy termination, offering valuable insights for reproductive health initiatives in Bangladesh and similar contexts.

## 1. Introduction

Reproductive health issues have emerged as one of the primary causes of mortality and disability among girls and women in developing nations. Unsafe pregnancy termination significantly compounds the challenges faced by mothers, elevating both maternal mortality and morbidity rates. Consequently, nonalcoholic fatty liver disease (NAFLD) has become a critical public health issue in numerous developing countries.^[1,2]^ The termination of pregnancy encompasses one of the many complexities associated with reproductive health, where adverse pregnancy outcomes may result in stillbirths, miscarriages, or induced abortions among women of reproductive age.^[3,4]^ Many women in this age group may resort to various abortion methods when faced with the possibility of an unexpected pregnancy.^[5,6]^

Data collected from 2010 to 2014 shed light on the significant prevalence of unwanted or unplanned pregnancies worldwide, accounting for nearly 44% of all pregnancies.^[7]^ Abortion terminated approximately 55.7 million pregnancies during this period.^[8]^ Worryingly, almost half of these abortions (25.1 million) were unsafe and occurred predominantly in low-income countries.^[9]^ Estimates suggest that between 40 and 50 million abortions occur annually, which equates to approximately 125,000 abortions per day.^[10]^ Tragically, unsafe abortions contribute to a considerable proportion of maternal fatalities, accounting for between 4.7% and 13.2% each year.^[11]^ Additionally, induced abortions account for approximately 20% of all pregnancies that terminate globally annually.^[12]^ It is crucial to note that the majority of induced abortions conducted in developing nations pose significant risks to women’s health and well-being.

Bangladesh, a vibrant developing nation situated in South Asia, is home to a population of approximately 142 million individuals. However, various challenges persist, including a poverty rate of 33% and an array of health-related issues.^[13]^ Women of reproductive age comprise slightly more than 46% of the female population in Bangladesh, and gender equality is a pressing concern.^[14]^ Being predominantly Muslim, Bangladesh reflects patriarchal traditions that often curtail women’s freedom of movement, limiting their access to education and economic opportunities. As a result, their roles primarily revolve around childbearing, with minimal societal involvement beyond this domain.^[12]^ These patriarchal norms, especially in matters of family planning, such as spacing pregnancies, determining the total number of pregnancies, and addressing pregnancy termination, place significant constraints on women’s autonomy and decision-making within such societies.^[9]^ Startlingly, voluntary abortions in Bangladesh reach 730,000 annually, accounting for 18% of all pregnancies.^[15]^ Over time, the average number of children per woman has decreased from nearly 7 in the 1970s to an average of 3 currently.^[16,17]^ An insightful study highlighted that socioeconomic factor, including educational attainment, occupation, income, and access to modern amenities, play vital roles in shaping reproductive health outcomes.^[6]^

Extensive research has indicated that pregnancy termination in developing nations is closely associated with sociodemographic characteristics. Factors such as a woman’s age, marital status, education level, employment position, and number of children have significant impacts on reproductive health and increase the risk of pregnancy termination.^[18]^ These socioeconomic and demographic factors vary from one society to another, even within different regions of the same country. A study also highlighted that young married girls face greater vulnerability to multiple pregnancies, recurrent miscarriages, pregnancy termination, and delivery complications.^[6]^ Shockingly, by the age of 18, approximately one-third of married young females in Bangladesh have already become mothers or are currently pregnant.^[4]^ Among the various reasons for induced pregnancy termination, unintended pregnancy is one of the primary factors. People often perform induced abortion, defined as the deliberate termination of a pregnancy to reduce fertility while preserving fecundity,^[19]^ for multiple reasons. These include safeguarding the mother’s life, protecting her physical and mental well-being, or addressing social and economic concerns, such as avoiding early pregnancies.^[20]^

Despite the illegality of induced abortion in Bangladesh, women often find themselves resorting to informal and traditional methods to terminate unintended pregnancies.^[9]^ These methods include using herbs, plant roots, homeopathy, oral contraceptives, or even hot salt water.^[9]^ Unfortunately, this situation leads to numerous unsafe pregnancy terminations performed by untrained individuals in risky environments,^[21]^ resulting in potential dangers to maternal health and even loss of life.^[2]^ Understanding the underlying factors contributing to pregnancy termination is highly important for mitigating the adverse effects of this significant problem. Moreover, predicting pregnancy termination in advance can enable timely intervention. Machine learning techniques offer a potential solution to overcome conventional regression models through thorough experimentation and review. These methods are better at identifying intricate links and patterns within the data, leading to more precise predictions. This development highlights the value of machine learning in healthcare prediction tasks and raises the possibility of using it to improve clinical judgement in the context of abortion. Hybrid models have demonstrated superior performance when compared to classical approaches, resulting in more balanced accuracy. Therefore, in this study we intended to see: what sociodemographic factors are the most significant predictors of pregnancy termination in Bangladesh, and how may hybrid machine learning models be used to predict this outcome correctly?

## 2. Materials and methods

### 2.1. Data sources and sampling design

This study relied on data obtained from the Bangladesh Demographic and Health Survey (BDHS), which provided a robust foundation for our research. Specifically, our analysis incorporated data from 3 separate time points: 2011, 2014, and 2017 to 2018 Bangladesh Demographic and Health Surveys (BDHS).^[22–24]^ The DHS employs a rigorous and well-established sample design, primarily using a 2-stage probability sampling method based on the most recent census data. Using the probability proportional to size (PPS) approach, the first stage selects principal sample units (PSUs) within each stratum. This ensures that larger PSUs have a higher probability of being selected, maintaining representation from various population segments. The second stage then conducts a comprehensive household listing for each selected cluster. We then employ a systematic selection process with equal probability to select a predetermined number of households from the compiled list. This meticulous approach ensures the inclusion of diverse households in the survey, enabling a comprehensive understanding of the population under which pregnancy termination was predicted and exploring the models’ significant and most impacted features.

### 2.2. Study population

As a sampling frame, the survey used a list of enumeration areas (EAs) from the 2011 Population and Housing Census of the People’s Republic of Bangladesh, provided by the Bangladesh Bureau of Statistics (BBS). The survey’s principal sample unit (PSU) is an EA with an average of 120 homes.

### 2.3. Dependent variable

In our study, the dependent variable under investigation was the occurrence of pregnancy termination. This variable was categorized into 2 distinct categories: “Yes” to denote terminated pregnancies and “No” to indicate pregnancies that were not terminated. Within our analysis, the value of zero was assigned to indicate “Yes,” while one was assigned to represent “No.” That is,


$$\begin{array}{l} Pregnancy\;Termination=\;\left\{ \begin{array}{l} No=1\;Yes=0\; \end{array}\right. \end{array}$$

### 2.4. Independent variables

For our analysis, we selected 10 independent variables to explore their potential influence on pregnancy termination. These variables encompassed a range of factors, including age (in years), residence, division, wealth index, working status, body mass index (BMI), total children ever born, births in the last 5 years, and the number of living children. By considering these diverse attributes, we aimed to comprehensively examine the associations of these attributes with the occurrence of pregnancy termination and gain insights into the multifaceted nature of this phenomenon.

### 2.5. Data preprocessing

#### 2.5.1. Data cleaning

Before proceeding with the machine learning analysis, the missing values within the dataset were carefully addressed and cleansed. Our dataset, consisting of combined information from the 2011 BDHS, the 2014 BDHS, and the 2017 to 2018 BDHS, initially contained a total of 54,881 respondent samples. However, it is important to note that not all observations contained complete information. After filtering out observations with missing values, 46,471 samples remained that contained detailed information on pregnancy termination and other relevant characteristics.

### 2.6. Data balancing

Class imbalance, where samples from different classes are unevenly represented, poses a significant challenge.^[25]^ This issue has been recognized by researchers as a crucial problem that can impact the accuracy of machine learning tasks. When faced with a substantial imbalance in the distribution of output classes, addressing this issue becomes crucial, particularly when dealing with small datasets.^[26]^ Various approaches can be employed to address this challenge, including selecting appropriate evaluation metrics; resampling techniques (such as oversampling or undersampling); employing methods such as the synthetic minority oversampling technique (SMOTE); utilizing a balanced bagging classifier; adjusting thresholds; and optimizing parameter values through grid search. In our study, we utilized the SMOTE technique to balance the dataset, ensuring that all the classes were adequately represented. Subsequently, the analysis was conducted using this balanced dataset, and multiple machine learning methods were employed to effectively classify pregnancy termination.

### 2.7. Splitting of the data and model building

The dataset was divided into 3 sections for the purposes of training, validation, and testing. Initially, the complete dataset was divided into a temporary training set (80%) and a test set (20%). The 80% training set was then subdivided into 75% actual training data and 25% validation data. We utilized a variety of models and fitted them with the training data. Using the validation set, model tuning was then performed to determine the optimal hyperparameters. To ensure robustness, we utilized 10-fold cross-validation and grid search to fine-tune the models and identify the best hyperparameters. After constructing the final best model, the fitting procedure was repeated on the training set. In conclusion, we analyzed the performance of the model using an independent test set, which provided useful insights into the prediction accuracy of our approach for pregnancy termination classification.

### 2.8. Feature selection

**Figure 1.**
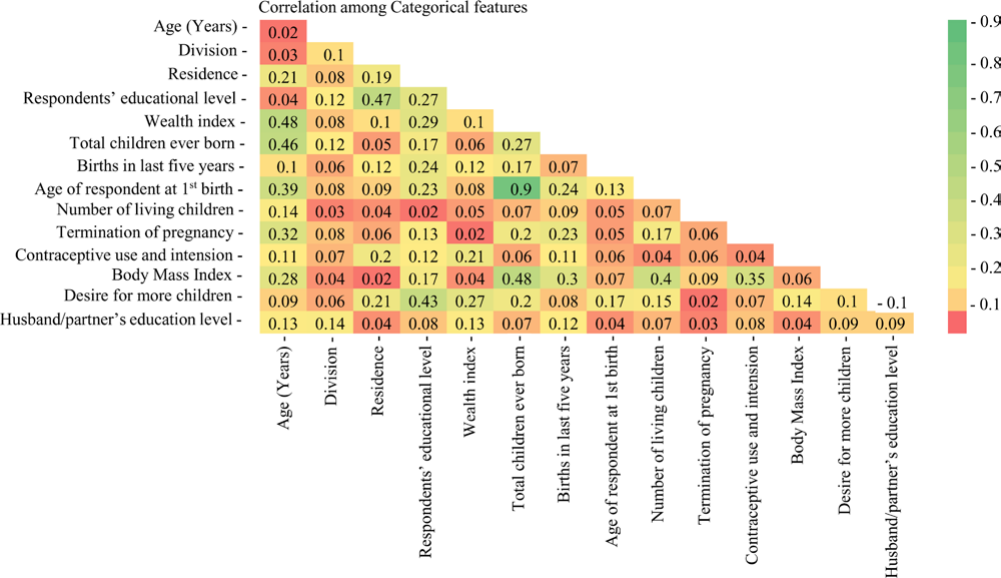
Correlations among categorical features.

First, we performed a chi-square test to assess the dependency of the target variable on the independent features. We selected only the statistically significant features (*P* ≤ .05). Now, we must check the associations or correlations among independent features. Given that the variables encompassed 2 or more categories, we employed Cramer V correlation among the features, as depicted in Figure 1. We eliminated the variable “total children ever born” because of its strong correlation (*R* = 0.90) with “number of living children.”

**Figure 2.**
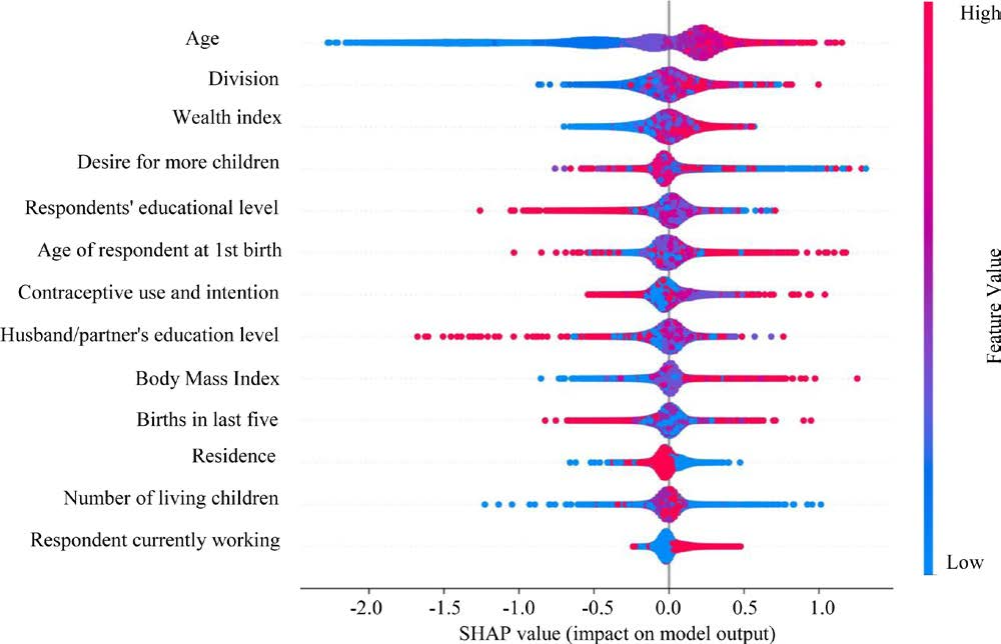
Impact of feature on the model.

Subsequently, we assessed the impact of the features on our model using XGBoost SHapley Additive Explanations (SHAP) values. The absolute SHAP value indicates how much each feature influences the prediction. Figure 2 presents the SHAP values and highlights the top ten features with the highest impact. In this figure, red indicates low feature magnitude, while blue indicates high feature magnitude. The horizontal axis at the bottom represents each feature’s contribution to the prediction. For instance, low age values contribute negatively to the prediction, while high values contribute positively. Similarly, for the division and wealth index, low values have a negative contribution, and high values have a positive one. However, these features show more density around the vertical line rather than to the left or right, which is why age has a greater impact on the model than the others. Moreover, features in the lower part of the figure primarily impact the vertical line without significantly affecting the positive or negative predictions. Therefore, we removed the features “resident,” “number of living children,” and “respondent currently working” from our model.

### 2.9. Statistical analysis

Upon retrieving the data, a meticulous validation process was undertaken to ensure the validity and reliability of the data. This involved discarding any missing, noneligible, or nonresponse values, ensuring that only high-quality data were utilized in our analysis. To gain a comprehensive understanding of the relationships between the independent and dependent variables, we employed the chi-square test for univariate analysis and conducted bivariate analysis to assess their associations. Subsequently, using the Jupyter notebook platform, we leveraged both traditional and hybrid machine learning techniques to predict pregnancy termination. It is worth noting that throughout these analyses, we considered the sample weights provided with the data, enabling us to appropriately account for the intricate sample design.

### 2.10. Machine learning (ML) approaches

In our study, we employed a comprehensive approach utilizing a combination of 8 classical machine learning models and hybrid machine learning models to predict pregnancy termination. The classical machine learning models include decision tree (DT), extreme gradient boosting (XGBoost), light gradient boosting machine (LightGBM), categorical boosting (CatBoost), K-nearest neighbors (KNN), random forest (RF), support vector machine (SVM), and adaptive boosting (AdaBoost) models. To evaluate the performance of these models, we employed various criteria, such as the area under the curve (AUC), precision, recall, and F1 score, to evaluate the results in both tabulated and graphical formats. The graphical presentations included a confusion matrix to illustrate the performance of the models, feature impact plots to highlight the significant predictors, area under the receiver operating characteristics (AUC–ROC) plots to show the AUC distribution of the models, and a precision–recall curve to determine the overall performance of the models.

### 2.11. Hybrid 1 (stacking)

The stacking ensemble algorithm, also known as stacked generalization, is a powerful machine learning technique. It utilizes a meta-learning algorithm to determine the optimal combination of predictions from multiple underlying machine learning algorithms. One of the key advantages of stacking is its ability to leverage the strengths of diverse and effective models, resulting in improved performance compared to that of any single model within the ensemble. This approach involves employing techniques such as bagging and boosting to integrate predictions from various machine learning models on the same dataset. The architecture of a stacking model consists of 2 or more base models, often referred to as level-0 models. These base models generate predictions that are subsequently fed into a meta-model, or level-1 model, which combines and integrates these predictions to yield the final output.

- Level-0 models (base models): Models fitted to training data whose predictions are compiled.
- Level-1 model (meta-model): This model discovers the ideal way to predict fundamental models.

The meta-model, often characterized by simplicity, plays a significant role in interpreting the predictions of base models. In our study, where the objective was classification, we opted for a meta-model in the form of logistic regression, and RF, XGBoost, and AdaBoost were used as base models.

### 2.12. Hybrid 2 (voting)

Voting is a collective machine learning algorithm that facilitates decision-making by combining the predictions of multiple models. In the context of regression tasks, a voting ensemble computes forecasts by averaging the predictions from various regression models. For classification tasks, 2 common approaches are employed: hard voting and soft voting. In hard voting, the ensemble aggregates the votes from different models for each distinct class label, ultimately predicting the class label that receives the highest number of votes. On the other hand, soft voting considers the predicted probabilities for each class label and selects the class label with the highest cumulative probability. This approach is based on summing the predicted probabilities from the various models. In our research, which focused on classification, we utilized a classification voting ensemble and combined the RF, XGBoost, and SVM models with a hard voting technique.

### 2.13. Ethical review

This study used a secondary data collected by NIPORT, Bangladesh and MEASURE DHS. All procedures performed in this study involving human participants were in accordance with the ethical standards of the national research committee and with the 1964 Helsinki Declaration and its later amendments or comparable ethical standards. As the data are freely available in the website, ethical review and approval was not required for the study on human participants in accordance with the local legislation and institutional requirements.

## 3. Results

### 3.1. Sociodemographic characteristics of the respondents

Table 1 presents the frequency distributions of various factors and their associations with pregnancy termination. Our findings revealed that 27% of women aged between 40 and 44 years had a greater likelihood of terminating pregnancy. Additionally, the termination rate is more prevalent in urban areas, where it accounts for 22.8% of the cases. The Sylhet division is associated with a greater tendency toward pregnancy termination, reaching 23%. Notably, the termination rate is notably elevated among the wealthiest women, constituting 22.4% of the cases. Among nonworking women, 69.4% had a significantly greater rate of pregnancy termination. When examining BMI, we observed that 57.5% of women in the normal weight category chose to terminate their pregnancies. Furthermore, our survey identified a notable proportion of women who had not given birth in the last 5 years, indicating a greater prevalence of pregnancy termination in this group. Notably, 41.7% of women who had more than 3 children ever born had a greater tendency toward pregnancy termination. Additionally, a significant majority of women (37%) with more than 3 living children also exhibited a higher rate of pregnancy termination. It is worth noting that among women who no longer desired to have more children, the incidence of pregnancy termination was particularly notable at 56.6%. The observed relationships between these selected factors and pregnancy termination were statistically significant (*P* < .01), underscoring their importance in influencing the occurrence of pregnancy termination.

**Table 1:** Frequency distribution of the factors and association with pregnancy termination (N = 51452).

Factors	Categories	Termination of pregnancy		$\chi^{2}$	*P* value
		No (%)	Yes (%)		
Age (yr)	15 to 19	5220 (92.4)	428 (7.6)	1293.95	<.01
	20 to 24	8226 (85.3)	1416 (14.7)		
	25 to 29	7741 (79.3)	2021 (20.7)		
	30 to 34	6450 (75.0)	2155 (25.0)		
	35 to 39	5087 (73.3)	1849 (26.7)		
	40 to 44	4301 (73.0)	1588 (27.0)		
	45 to 49	3636 (73.2)	1334 (26.8)		
Residence	Urban	13,888 (77.2)	4109 (22.8)	57.689	<.01
	Rural	26,773 (80.0)	6682 (20.0)		
Division	Barisal	4593 (78.7)	1240 (21.3)	55.315	<.01
	Chittagong	6506 (81.7)	1453 (18.3)		
	Dhaka	8260 (79.3)	2160 (20.7)		
	Khulna	5747 (78.9)	1538 (21.1)		
	Rajshahi	5638 (78.6)	1537 (21.4)		
	Rangpur	5458 (78.1)	1529 (21.9)		
	Sylhet	4459 (77.0)	1334 (23.0)		
Wealth index	Poorest	7541 (81.4)	1718 (18.6)	94.074	<.01
	Poorer	7763 (79.8)	1969 (20.2)		
	Middle	8089 (79.5)	8089 (20.5)		
	Richer	8503 (78.8)	2282 (21.2)		
	Richest	8765 (76.2)	2741 (23.8)		
Working status	Yes	12,112 (76.9)	3639 (23.1)	62.158	<.01
	No	28,549 (80.0)	7152 (20.0)		
Body mass index	Under weight	7249 (82.2)	1573 (17.8)	200.861	<.01
	Normal weight	23,599 (79.8)	5987 (20.2)		
	Over weight	8024 (76.1)	2520 (23.9)		
	Obese	1789 (71.6)	711 (71.6)		
Total children ever born (No.)	0	4395 (88.4)	575 (11.6)	556.232	<.01
	1	9164 (83.2)	1849 (16.8)		
	2	10,925 (77.8)	3112 (22.2)		
	3+	16,177 (75.5)	5255 (24.5)		
Births in last 5 years (No.)	0	23,379 (76.9)	7042 (23.1)	214.762	<.01
	1	14,501 (82.0)	3185 (18.0)		
	2+	2781 (83.1)	564 (16.9)		
Number of living children	0	4601 (87.8)	642 (12.2)	507.450	<.01
	1	9750 (82.7)	2040 (17.3)		
	2	11,958 (77.8)	3409 (22.2)		
	3+	14,352 (75.3)	4700 (24.7)		

### 3.2. Performance measure of the classifier

We evaluated different machine learning algorithms on our dataset considering 4 essential measurement criteria, AUC, precision, recall, and F1 score, since the data were imbalanced in frequency, which is why we did not consider the accuracy of the models. Based on the findings presented in Table 2, the Hybrid 2, SVM, Hybrid 1, RF, XGBoost, AdaBoost, KNN, CatBoost, LightGBM, and DT algorithms achieved AUSs of 91.97%, 82.81%, 80.91%, 80.44%, 80.04%, 78.23%, 77.93%, 77.33%, 77.11%, and 71.19%, respectively. Notably, the Hybrid 2 algorithm exhibited superior performance across all the measurement criteria, as it had the highest AUC, precision, recall, and F1 score. Conversely, DT had the lowest values in terms of AUC, precision, recall, and F1 score. Overall, in regard to the prediction capabilities for our dataset, the Hybrid 2 technique outperformed the other 9 algorithms, demonstrating its effectiveness and suitability for our specific data analysis task.

**Table 2:** Evaluation of the model performance.

Models	AUC	Precision	Recall	F1 score
Hybrid 2	91.97	84.14	83.87	83.84
SVM	82.81	75.68	75.45	75.41
Hybrid 1	80.91	73.62	73.59	73.59
RF	80.44	73.07	72.77	72.71
XGBoost	80.04	73.17	73.02	72.99
AdaBoost	78.23	72.99	72.75	72.70
KNN	77.93	71.96	70.55	70.11
CatBoost	77.33	72.28	72.06	72.01
LightGBM	77.11	70.67	70.42	70.34
DT	71.19	67.03	66.55	66.36

AdaBoost = adaptive boosting, AUC = area under the curve, CatBoost = categorical boosting, DT = decision tree, KNN = K-nearest neighbors, LightGBM = light gradient boosting machine, XGBoost = extreme gradient boosting.

### 3.3. AUC–ROC curves of the classifiers

**Figure 3.**
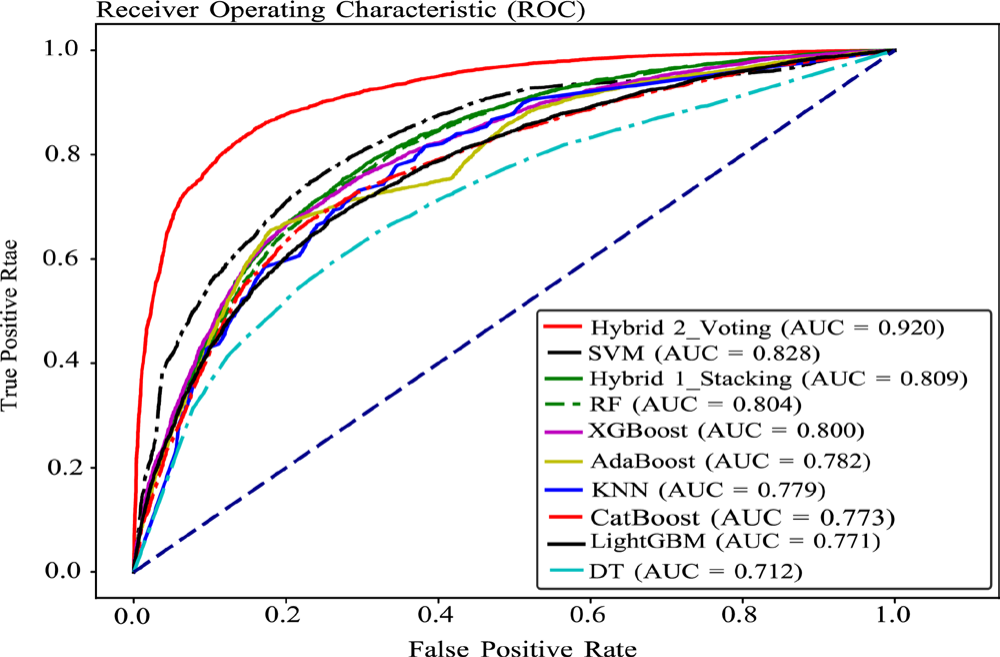
AUC–ROC curve of the models.

The AUC–ROC is an appropriate statistic for imbalanced datasets and binary classification tasks because it considers the trade-off between sensitivity (true positive rate) and specificity (true negative rate) at different probability thresholds. A value of 0.5 for the AUC suggested random guessing, whereas 1.0 indicated flawless categorization. The closer the AUC is to 1, the greater the discrimination ability of the model. The AUC–ROC values of our models demonstrated how well each model could distinguish between 2 pregnancy outcome groups (termination and nontermination). Based on the AUC values of the models, Hybrid 2 fared better than the other models did, while DT performed worse. The SVM, Hybrid 1, RF, and XGBoost algorithms performed nearly the same (Fig. 3).

### 3.4. Precision–recall curves of the models

**Figure 4.**
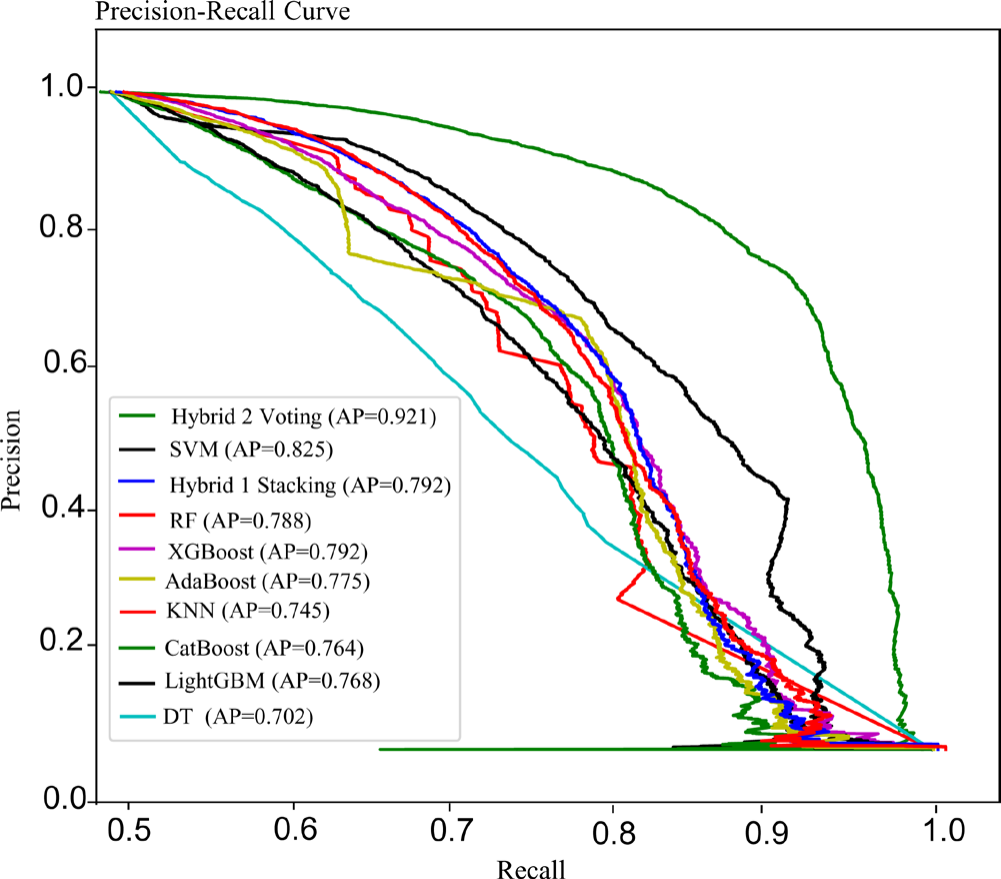
Precision–recall curve.

Precision–recall curves (Fig. 4) illustrate the trade-off between precision (positive predictive value) and recall (true positive rate) for different classification thresholds. Like in the ROC curves, higher precision and recall values closer to 1 are preferred. The average precision summarizes the trade-off between precision and recall across all potential classification levels. It delivers a single scalar value that indicates the model’s overall performance in terms of precision and recall. Based on our average precision values, Hybrid 2 outperformed the competition again, while DT received the lowest score.

### 3.5. Most important features

**Figure 5.**
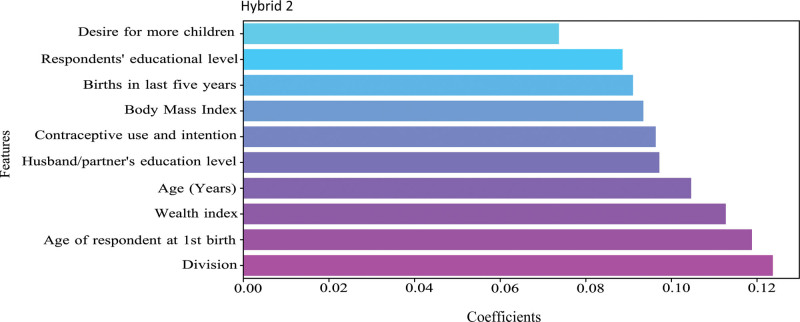
Feature importance of the Hybrid 2 model.

The feature importance of the Hybrid 2 model is presented in Figure 5. Division, age of respondent at first birth, wealth index, and age are the most important features for predicting pregnancy termination according to our best model, Hybrid 2.

## 4. Discussion

Our study revealed a range of compelling features that exhibited significant associations with pregnancy termination. Among these influential factors, age emerged as a crucial variable, shedding light on how different age groups may be more prone to opting for pregnancy termination. Moreover, the residence and division variables played a notable role, highlighting potential regional disparities and contextual factors that influence women’s decisions regarding pregnancy termination. The wealth index variable revealed intriguing insights into the impact of socioeconomic status, suggesting that financial considerations may influence the likelihood of pregnancy termination. Furthermore, working status emphasizes the relationship between employment and reproductive choices. BMI emerged as a significant factor, indicating a potential relationship between maternal health and pregnancy termination decisions. The variables capturing the total number of children ever born, births in the last 5 years, and the number of living children highlighted the complexities associated with family dynamics and reproductive choices.

In 2019, a noteworthy study explored the prediction of pregnancy termination using various machine learning classifiers, including naive Bayes, decision jump, Bayesian network, and hybrid models. Interestingly, the findings of this study resonate with our own research, as they identified the hybrid classifier as the superior performer among the different models. Specifically, the hybrid classifier demonstrated an accuracy of 80.60% in our analysis, surpassing the reported accuracy of 67.20% from the literature.^[27]^ A crucial factor contributing to the enhanced accuracy in our study was the meticulous parameter tuning we carried out prior to employing the diverse machine learning classifiers. It is well known that parameter tuning plays a significant role in fine-tuning models and optimizing their performance, thereby improving accuracy.^[28]^ Thus, careful consideration and adjustment of these parameters positively influenced the outcomes of our analysis, leading to a more accurate prediction of pregnancy termination.

In our analysis, an intriguing finding emerged regarding the association between the number of living children and pregnancy termination. Our results contradicted Bairagi 2001 study,^[29]^ showing that women with more than 3 living children were more likely to choose pregnancy termination. Interestingly, Bairagi study highlighted that women without any living sons were less inclined to terminate pregnancy, whereas women who had already given birth to a son were more likely to terminate the pregnancy.^[30]^ The National Institute of Population Research and Training (NIPORT) conducted a separate study which revealed that women over 30 who had 2 or more children were more inclined to terminate their pregnancies.^[31]^ Remarkably, our findings align with the conclusions of the NIPORT report, reinforcing the relationship between age, number of children, and the propensity for pregnancy termination.

The findings of this study also demonstrated that the Hybrid 2 model achieved favorable results in terms of balanced outcomes for the 2 classes, surpassing the performance of the other models. Notably, when considering the rates of true positive and true negative predictions, the SVM demonstrated 63% to 77%, Hybrid 1 71% to 75%, and Hybrid 2 79% to 82%. A recent study conducted in 2022 utilized a hybrid ensemble algorithm, achieving an impressive accuracy of 93.99%, a recall of 92.44%, and a precision of 93.46%, thus outperforming other machine learning models.^[32]^ Comparatively, our study revealed that the Hybrid 2 model performed better than both classical methods and other machine learning classifiers, making it the best model. The Hybrid 2 model displayed balanced predictions for both classes, minimizing the imbalance between results and delivering satisfactory accuracy. Conversely, the SVM model exhibited the poorest performance in terms of imbalance. Notably, wealth index, division, and age emerged as the most significant features across multiple models, indicating their importance in predicting pregnancy termination.

Several recent studies have utilized machine learning algorithms and national health survey data to predict various health outcomes. In 2022, one study focused on forecasting cesarean births in Bangladesh, employing logistic regression and 5 machine learning algorithms to evaluate accuracy, precision, recall, and F1 score.^[33]^ Our study, however, emphasized hybrid models, specifically highlighting metrics such as AUC, accuracy, recall, and F1. Both studies identified significant sociodemographic factors, with the former highlighting their statistical importance and socioeconomic disparities in cesarean rates. In 2024, researchers conducted another study that used primary data and KNN, RF, and CatBoost models to predict suicidal ideation among Bangladeshi students.^[34]^ Both studies examine important public health issues and identify key factors; however, they may differ in terms of focus and data used.

### 4.1. Policy implementation

The results of this investigation have significant social implications not only for Bangladesh, but also for other countries grappling with similar challenges related to pregnancy termination. The identification of sociodemographic factors associated with this issue can help policymakers and healthcare providers devise targeted interventions and formulate effective policies aimed at reducing its prevalence. The study findings indicate that women aged 40 to 44 years, urban dwellers, and those with higher socioeconomic status exhibit higher rates of pregnancy termination. This highlights the need for tailored interventions that specifically address the underlying reasons contributing to the elevated levels within these particular groups. Such interventions could include improving access to family planning and contraceptive services, increasing the availability of high-quality maternal and reproductive healthcare, and tackling socioeconomic inequalities that contribute to pregnancy termination. Furthermore, the study emphasizes the significance of considering regional disparities and the unique needs of people of various ages and socioeconomic groups when designing interventions. Notably, the identification of division, age of respondent at first birth, wealth index, and age as the most crucial predictors of pregnancy termination underscores the importance of addressing these factors in endeavors to reduce its prevalence. Taking a comprehensive approach to addressing pregnancy termination is crucial, as evidenced by this study. By figuring out the main societal and demographic factors that affect this issue and using machine learning to predict pregnancy termination, policymakers and healthcare providers can come up with more targeted interventions and policies. This will eventually lead to a drop in the number of abortions and a rise in the health and well-being of women and their families around the world, not just in Bangladesh.

### 4.2. Strengths and limitations

This research presented several commendable aspects, including its use of representative data that accurately represent the entire female population of Bangladesh, enabling broad conclusions to be drawn. Notably, this was the first study to incorporate both Hybrid Model 1 (stacking) and Hybrid Model 2 (voting), which are noteworthy contributions to the field. However, there are certain limitations that should be acknowledged. The data employed in the research spanned from 2011 to 2017 to 2018, as no updated demographic data were available for the nation at the time. Consequently, these findings may not precisely reflect the current sociodemographic characteristics of Bangladeshi women from 2021 to 22. Furthermore, importantly, this study did not explore all possible combinations of hybrid machine learning models, leaving room for further investigation in this area.

## 5. Conclusion

This study presents significant findings regarding the application of machine learning in reproductive health research. Notably, it was observed that Hybrid Model 2 outperformed traditional methods and other machine learning classifiers, exhibiting balanced results and satisfactory accuracy. This study highlights the alarmingly high rate of pregnancy termination among respondents, which can be mitigated by focusing on associated factors. The identification of age, division, and wealth index as the most influential features in predicting pregnancy termination underscores their importance. Policymakers should prioritize efforts to reduce poverty, enhance job opportunities, and provide support to individuals with low incomes, aiming to address socioeconomic inequities. Recognizing the diverse needs of urban and rural women, tailored interventions may be necessary to combat this issue effectively and promote the health and well-being of women and their families in Bangladesh and similar contexts.

## Author contributions

**Conceptualization:** Riaz Rahman, Ashis Talukder.

**Data curation:** Riaz Rahman, Ashis Talukder, Shatabdi Das.

**Formal analysis:** Riaz Rahman, Shatabdi Das.

**Methodology:** Riaz Rahman, Ashis Talukder, Shatabdi Das, Joy Saha.

**Software:** Riaz Rahman, Joy Saha.

**Writing – original draft:** Riaz Rahman, Ashis Talukder, Shatabdi Das, Joy Saha, Haribondhu Sarma.

**Writing – review & editing:** Riaz Rahman, Ashis Talukder, Shatabdi Das, Joy Saha, Haribondhu Sarma.

**Investigation:** Ashis Talukder.

**Supervision:** Ashis Talukder.

**Validation:** Haribondhu Sarma.

**Visualization:** Haribondhu Sarma.
